# Effectiveness of Sterile Versus Clean Indwelling Catheter Care in Preventing Catheter-Associated Urinary Tract Infections at a Tertiary Care Center: A Randomized Controlled Trial

**DOI:** 10.7759/cureus.96540

**Published:** 2025-11-11

**Authors:** Ankur Mittal, Deepesh Swami, Vikas K Panwar, Balram J Omar, Kunal Malhotra, Kanta Bairwa, Jayakrishna Sahu, Arup Mandal

**Affiliations:** 1 Urology, All India Institute of Medical Sciences, Rishikesh, IND; 2 Nursing, All India Institute of Medical Sciences, Rishikesh, IND; 3 Microbiology, All India Institute of Medical Sciences, Rishikesh, IND

**Keywords:** catheter-associated urinary tract infection, clean technique, indwelling urinary catheter, nursing care, resource-limited settings, sterile technique

## Abstract

Background

Catheter-associated urinary tract infection (CAUTI) is a leading hospital-acquired infection, contributing to increased morbidity and healthcare costs. While sterile catheter care is standard, major guidelines recommend clean maintenance care, though debate continues.

Objective

The objective of the study is to compare the effectiveness of sterile versus clean indwelling catheter care in preventing CAUTI.

Methods

A participant-blind, computer-generated, stratified block randomized controlled trial was conducted at a tertiary care center. Patients with sterile baseline urine cultures were randomized to either the sterile or clean catheter care group. Data collection continued until catheter removal, hospital discharge with catheter in situ, Day 14 of catheterization, or onset of CAUTI symptoms, whichever occurred first. CAUTI symptoms including fever > 38°C, rigors, pus discharge, suprapubic pain, agitation, and confusion were monitored. The pre-specified non-inferiority margin was 12%. Analyses were performed per protocol; no intention-to-treat analysis was conducted.

Results

Among 346 participants, CAUTI occurred in 7.5% of the clean group and 6.9% of the sterile group (p = 0.83). Group assignment was not associated with CAUTI in adjusted analysis, while catheter duration was the strongest independent predictor (adjusted odds ratio (aOR): 1.262, 95% confidence interval (CI): 1.135-1.400). The overall incidence was 20.64 per 1,000 catheter days, with a median catheterization duration of four days in the clean group and three days in the sterile group. *Pseudomonas*, *Klebsiella*, and *Escherichia coli* were the most common isolates, with some showing multidrug resistance. The study was underpowered to detect small between-group differences.

Conclusion

Clean catheter care was comparable to sterile care in this single-center trial. While it may be a practical alternative, conclusions are limited by underpowering and methodological constraints. Larger multicenter studies with intent-to-treat analyses and adjustment for antibiotic exposure are needed to confirm these findings.

## Introduction

Catheter-associated urinary tract infection (CAUTI) is one of the most prevalent nosocomial infections, accounting for approximately 30-40% of hospital-acquired infections worldwide and nearly 9% of all healthcare-associated infections [1,2]. The risk of bacteriuria increases by 3-10% per day of catheterization, and 10-25% of catheterized patients develop bacteriuria, with a subset progressing to symptomatic CAUTI [3]. Prolonged catheterization remains the most important modifiable risk factor, underscoring the need for optimized catheter care protocols.

While sterile insertion of indwelling catheters is universally recommended [4], the superiority of sterile over clean maintenance care remains debated. Major guidelines including the CDC, AHRQ, and recent expert consensus recommend clean maintenance care for indwelling catheters, acknowledging that sterile care is resource-intensive and not routinely required [1,4,5]. Clean catheter care is less resource-intensive and has been widely adopted for home-based [6] and long-term management in Western countries [7,8]. Small-scale studies, such as the one conducted at PGIMER Chandigarh, have reported no statistically significant differences in CAUTI incidence between sterile and clean techniques, suggesting potential equivalence if procedures are performed correctly [3]. However, the limited sample sizes and single-center designs reduce the generalizability of these findings, and robust hospital-based evidence remains insufficient.

The rising prevalence of antimicrobial resistance (AMR) further complicates CAUTI management. Multidrug-resistant (MDR) pathogens such as extended-spectrum β-lactamases (ESBLs) producing Enterobacterales, carbapenem-resistant Klebsiella, and fluoroquinolone-resistant Pseudomonas are increasingly reported in hospital-acquired urinary tract infections (UTIs) [9-13]. Studies have shown that inappropriate antibiotic use accelerates the emergence of resistant strains, highlighting the need for preventive strategies that reduce catheter days and optimize catheter care to minimize both CAUTI incidence and antimicrobial overuse [12-15].

Evidence-based strategies to prevent CAUTI include aseptic insertion of catheters, maintaining a closed drainage system, performing appropriate catheter hygiene, and daily reassessment of catheter necessity [16-18]. Despite these recommendations, the comparative effectiveness of sterile versus clean catheter care in hospital practice remains underexplored.

Study objectives

The primary objective was to assess the effectiveness of sterile versus clean indwelling catheter care techniques in preventing CAUTI among hospitalized adult patients in a tertiary care setting. The secondary objectives were to (1) identify factors associated with CAUTI, (2) determine CAUTI rates per 1,000 catheter-days, and (3) analyze the antibiogram profiles of the isolated pathogens.

We hypothesized that the clean technique would be non-inferior to the sterile technique in hospital settings and, if demonstrated, could provide a sustainable, cost-effective alternative for both hospitalized patients and long-term catheter users in resource-limited settings [19-21].

## Materials and methods

Participants

Inclusion criteria were: (1) adult patients undergoing Foley catheterization admitted at a tertiary care center, (2) catheterization within the past 24 hours, including those catheterized in other areas, and (3) sterile baseline urine culture (sample collected during insertion or within the first 24 hours).

**Figure 1:**
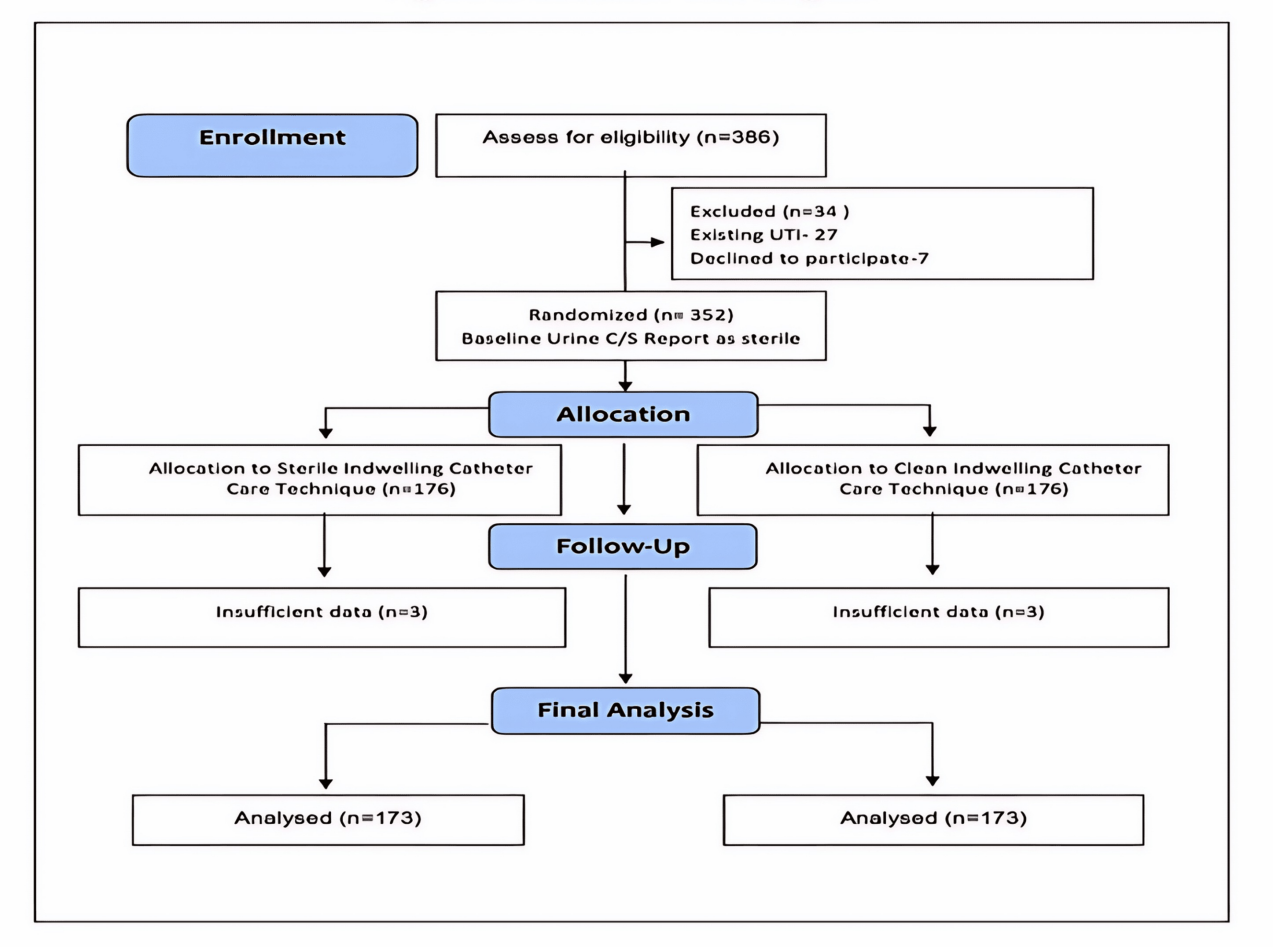
CONSORT flow diagram CONSORT: Consolidated Standards of Reporting Trials

Exclusion criteria were (1) pregnancy, (2) uncontrolled diabetes, (3) existing UTI, (4) known latex allergy, and (4) immunocompromised status (Figure 1). Written informed consent was obtained from all participants in the language they best understood. 

The study protocol was approved by the Research Cell and Institutional Ethics Committee (Approval No.: AIIMS/IEC/22/436, dated 05-08-2022) and was registered with the Clinical Trials Registry of India (CTRI/2022/11/047662) on 25th November 2022.

Study design and randomization

This was a single-center, parallel-arm randomized controlled trial. Participants were randomized into sterile and clean catheter care groups using a computer-generated allocation sequence stratified by age (<50 years and ≥50 years) in a 1:1 ratio. Allocation concealment was maintained using the Sequentially Numbered, Opaque Sealed Envelope (SNOSE) method. Sequentially numbered envelopes were opened in order only after confirming participant eligibility and sterile baseline urine culture. Baseline urine culture and sensitivity testing was performed for all participants, ensuring sterile urine culture at enrolment.

Blinding

This was a single-blind randomized controlled trial. Participants were informed they would receive one of two accepted catheter care approaches but were not told which one they were assigned to. A bedside cardiac table was used to hold supplies and block direct vision of the procedure, thereby maintaining participant blinding. Blinding of healthcare providers performing catheter care was not feasible due to the nature of the intervention; this has been acknowledged as a methodological limitation. Microbiologists analysing urine cultures were blinded to group assignment.

Intervention

The sterile technique used sterile gloves, gauze, artery forceps, and strict aseptic precautions. The clean technique followed standardized protocols using clean gloves and gauze. Normal saline (0.9%), supplied by the hospital pharmacy, was used for urethral meatus and catheter tubing cleansing. Catheter care was performed once daily, preferably in the morning. The detailed stepwise protocols for both sterile and clean catheter care techniques were developed by the research team based on international guidelines and institutional recommendations (Supplementary Appendix 1).

Monitoring and fidelity

CAUTI symptoms (fever >38°C, rigors, pus discharge, suprapubic pain/tenderness, agitation, confusion) were monitored daily by core research team members. A follow-up urine culture was obtained if symptoms developed or at predefined time points: (1) catheter removal, (2) hospital discharge with catheter in situ, or (3) the 14th day of catheterization. Significant bacteriuria was defined as ≥10^5 CFU/ml. Antibiotics were prescribed based on clinical indications by the treating consultant.

Before the study, catheterization sets were prepared in the Urology Department. Nursing officers and residents were trained on CDC guidelines for sterile catheterization [1], urine specimen collection, SOPs, and both sterile and clean catheter care techniques. Regular Continuing Nursing Education workshops reinforced adherence. Catheter care in both groups was delivered by the same pool of nursing officers. To promote fidelity, members of the research team conducted unannounced spot checks of catheter insertions and care procedures, ensuring adherence to assigned protocols. Overall adherence to the stepwise protocol during spot checks was consistently high, and any observed deviations were immediately corrected through on-site feedback. The potential for a Hawthorne effect was recognized and minimized through routine supervisory monitoring.

Microbiological analysis

Urine specimens were collected aseptically from the sampling port using a sterile syringe and container, after disinfecting with 70% isopropyl alcohol, in accordance with NHS guidelines [22] (Supplementary Appendix 2). Samples were transported to the microbiology laboratory within two hours of collection.

Organisms were identified using conventional biochemical methods and automated systems (VITEK/Microscan). Antimicrobial susceptibility testing (AST) was conducted using automated systems that generated minimum inhibitory concentration (MIC) values for each antimicrobial agent, which were interpreted according to standard CLSI breakpoints. Colistin testing was performed by automated methods, with confirmatory broth microdilution where indicated.

AST results were reported using a cascade reporting strategy, in which Tier 1 (primary) agents were preferentially reported, and higher-tier agents (Tiers 2-4) were reported only when resistance to primary drugs was detected, consistent with institutional antimicrobial stewardship policy. Final results were expressed as Sensitive (S), Intermediate (I), Resistant (R), or Susceptible Dose Dependent (SDD). Multidrug resistance was defined as resistance to at least one agent in three or more antimicrobial classes, and ESBL production was confirmed using the combination disc method.

Outcomes

The primary outcome was the incidence of CAUTI in clean versus sterile care groups. CAUTI was defined as ≥10⁵ CFU/mL of one or more pathogens in urine culture, with at least one clinical feature of infection (fever >38°C, suprapubic pain/tenderness, rigors, agitation, or purulent discharge). Asymptomatic bacteriuria was not considered CAUTI. Definitions were aligned with CDC criteria. Secondary outcomes were: (1) factors associated with CAUTI, (2) CAUTI rate per 1,000 catheter days, and (3) the microbiological flora and antibiogram of urine culture isolates.

Statistical analysis

Data were entered in Microsoft Excel and analysed using IBM SPSS Statistics for Windows, Version 29 (Released 2023; IBM Corp., Armonk, New York, United States). The study assessed whether clean catheter care was comparable to sterile catheter care, using a pre-specified non-inferiority margin of 12%. A Z-test determined the required sample size of 176 participants per group (total 352) at 80% power and a 5% type I error rate, accounting for an anticipated 10% attrition. Between-group differences in CAUTI incidence were analysed using the chi-square (χ²) test. Binary logistic regression was performed to identify factors associated with CAUTI. Analyses were performed per protocol; an intent-to-treat analysis was not conducted. Antibiotic exposure was prescribed as per clinical indications by the treating consultant but could not be consistently recorded across participants; therefore, it was not included as a covariate in regression models. Antibiogram data were summarized descriptively as MIC values and categorized as Sensitive (S), Intermediate (I), or Resistant (R) according to CLSI breakpoints. All statistical tests were two-tailed, and p < 0.05 was considered statistically significant. Ninety-five percent confidence intervals (95% CI) were calculated for proportions and odds ratios (OR).

## Results

The study enrolled 346 participants, equally divided into two groups of 173 each. Baseline demographic and clinical characteristics, including age, gender, catheter size and type, random blood sugar (RBS), and duration of catheterization, were approximately similar between groups (Table 1). A small difference was noted in the proportion of patients discharged from the hospital with the catheter still in situ (6.4% in the clean group vs. 12.7% in the sterile group), though this was not statistically significant.

**Table 1:** Baseline Characteristics of Participants This table presents the baseline demographic and clinical characteristics of participants randomized to clean (n=173) and sterile (n=173) catheter care. Variables include age (median with interquartile range), age group distribution, gender, catheter size (in French), catheter type (double vs. triple lumen), random blood sugar (mean ± standard deviation), duration of catheterization (median with interquartile range), and discharge status with catheter in situ. No statistically significant differences were observed between groups, confirming comparability at baseline. IQR: Interquartile Range; PUC: Per Urinary Catheter; RBS: Random Blood Sugar

Characteristic	Clean Group (n=173)	Sterile Group (n=173)	Total (n=346)
Age (Years), Median (IQR)	51 (33-60)	50 (36-63)	50 (34-61)
Age Group, n (%)			
Below 50 Years	87 (50.3%)	87 (50.3%)	174 (50.3%)
50 Years and Above	86 (49.7%)	86 (49.7%)	172 (49.7%)
Gender, n (%)			
Male	138 (79.8%)	136 (78.6%)	274 (79.2%)
Female	35 (20.2%)	37 (21.4%)	72 (20.8%)
Size of PUC (Fr.), n (%)			
12	2 (1.2%)	0 (0.0%)	2 (0.6%)
14	21 (12.1%)	22 (12.7%)	43 (12.4%)
16	71 (41.0%)	79 (45.7%)	150 (43.4%)
18	14 (8.1%)	17 (9.8%)	31 (9.0%)
20	2 (1.2%)	1 (0.6%)	3 (0.9%)
22	63 (36.4%)	54 (31.2%)	117 (33.8%)
Type of PUC, n (%)			
Double Lumen	115 (66.5%)	124 (71.7%)	239 (69.1%)
Triple Lumen	58 (33.5%)	50 (28.3%)	108 (31.2%)
RBS, Mean ± SD	105.87 ± 21.66	109 ± 24.09	107.43 ± 22.96
PUC Days, Median(IQR)	4 (3-7)	3 (2-6)	4 (2-7)
Discharge on PUC, n (%)	11 (6.4%)	22 (12.7%)	33 (9.5%)

Primary outcome: CAUTI incidence

Among 173 patients in each arm, CAUTI occurred in 13 patients (7.5%) in the clean group and 12 patients (6.9%) in the sterile group (p = 0.83). The difference was not statistically significant, indicating no clear evidence of superiority of either technique. Given the relatively low number of CAUTI events, the study may have been underpowered to detect small between-group differences.

Factors associated with CAUTI

Univariate analysis revealed a significant association between catheterization duration and CAUTI. This association remained highly significant in multivariate analysis, confirming catheter duration as an independent predictor of CAUTI (aOR: 1.262, 95% CI: 1.135-1.400) (Table 2).

**Table 2:** Univariate and Multivariate Logistic Regression Analysis for Variables Affecting CAUTI This table summarizes the results of univariate and multivariate logistic regression analyses for factors associated with catheter-associated urinary tract infection (CAUTI). Variables assessed included age, catheter size, random blood sugar (RBS), duration of catheterization (PUC days), study group (clean vs. sterile), and catheter type (double vs. triple lumen). In the univariate analysis, longer duration of catheterization was significantly associated with CAUTI (OR: 1.250; 95% CI: 1.130–1.390; p<0.01). After adjustment, catheter duration remained an independent predictor of CAUTI (aOR: 1.262; 95% CI: 1.135–1.400; p<0.001). No other variables were statistically significant. OR: Odds Ratio; aOR: Adjusted Odds Ratio; CI: Confidence Interval; RBS: Random Blood Sugar; PUC: Per Urethral Catheter

Variables	Univariate Analysis		Multivariate Analysis	
	P-value	Odds Ratio (95% CI)	P-value	Adjusted Odds Ratio (95% CI)
Age in Years	0.54	0.990 (0.960–1.010)	0.92	1.002 (0.971–1.030)
Size of PUC	0.62	0.960 (0.840–1.100)	0.68	0.968 (0.827–1.130)
RBS	0.11	0.980 (0.960–1.010)	0.12	0.985 (0.966–1.010)
PUC Days	<0.01	1.250 (1.130–1.390)	<0.001	1.262 (1.135–1.400)
Clean vs. Sterile Group	0.83	1.090 (0.480–2.460)	0.77	1.135 (0.480–2.680)
Type of Catheter (Double vs. Triple)	0.33	1.250 (0.800–1.950)	0.33	1.250 (0.800–1.950)

Descriptive findings: symptom trends over time in catheterized patients

**Figure 2:**
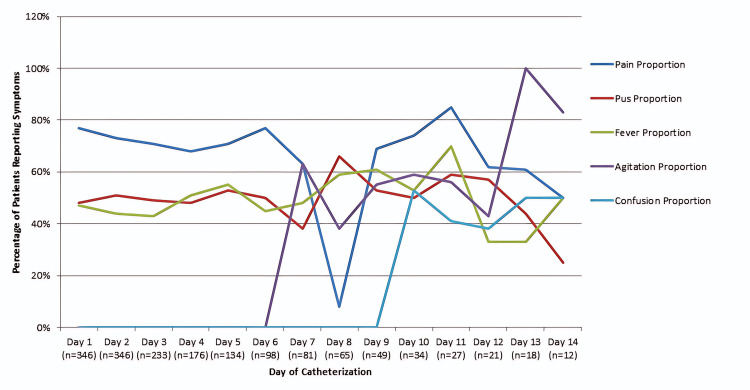
Symptom Monitoring Over 14 Days in Patients with PUC This figure illustrates the daily prevalence of catheter-associated symptoms during the 14-day catheterization period. Pain was the most frequent symptom, reported in 77% of patients on Day 1, declining to 50% by Day 14. Pus discharge peaked at 66% on Day 8 before declining to 25%. Fever fluctuated, with a maximum prevalence of 70% on Day 11 and decreasing to 50% by Day 14. Agitation first appeared on Day 7, peaked at 100% on Day 13, and subsequently declined, while confusion emerged on Day 10 and persisted in 50% of patients at Day 14. These data reflect overall symptom burden but should be interpreted with caution, as concurrent treatments such as analgesics and antipyretics were not standardized. PUC: Per Urethral Catheter

Daily monitoring showed variable symptom prevalence during the catheterization period. Pain was the most frequently reported symptom, present in 77% of patients on Day one and declining to 50% by Day 14. Pus discharge peaked at 66% on Day eight before reducing to 25%. Fever fluctuated, reaching 70% on Day 11 and decreasing to 50% by Day 14. Agitation first appeared on Day seven, peaked at 100% on Day 13, and subsequently declined, while confusion emerged on Day 10 and persisted in 50% of patients at Day 14 (Figure 2). These findings describe the overall symptom burden over time but should be interpreted with caution, as concurrent treatments such as analgesics and antipyretics were not standardized or recorded.

Descriptive findings: suprapubic pain intensity

**Figure 3:**
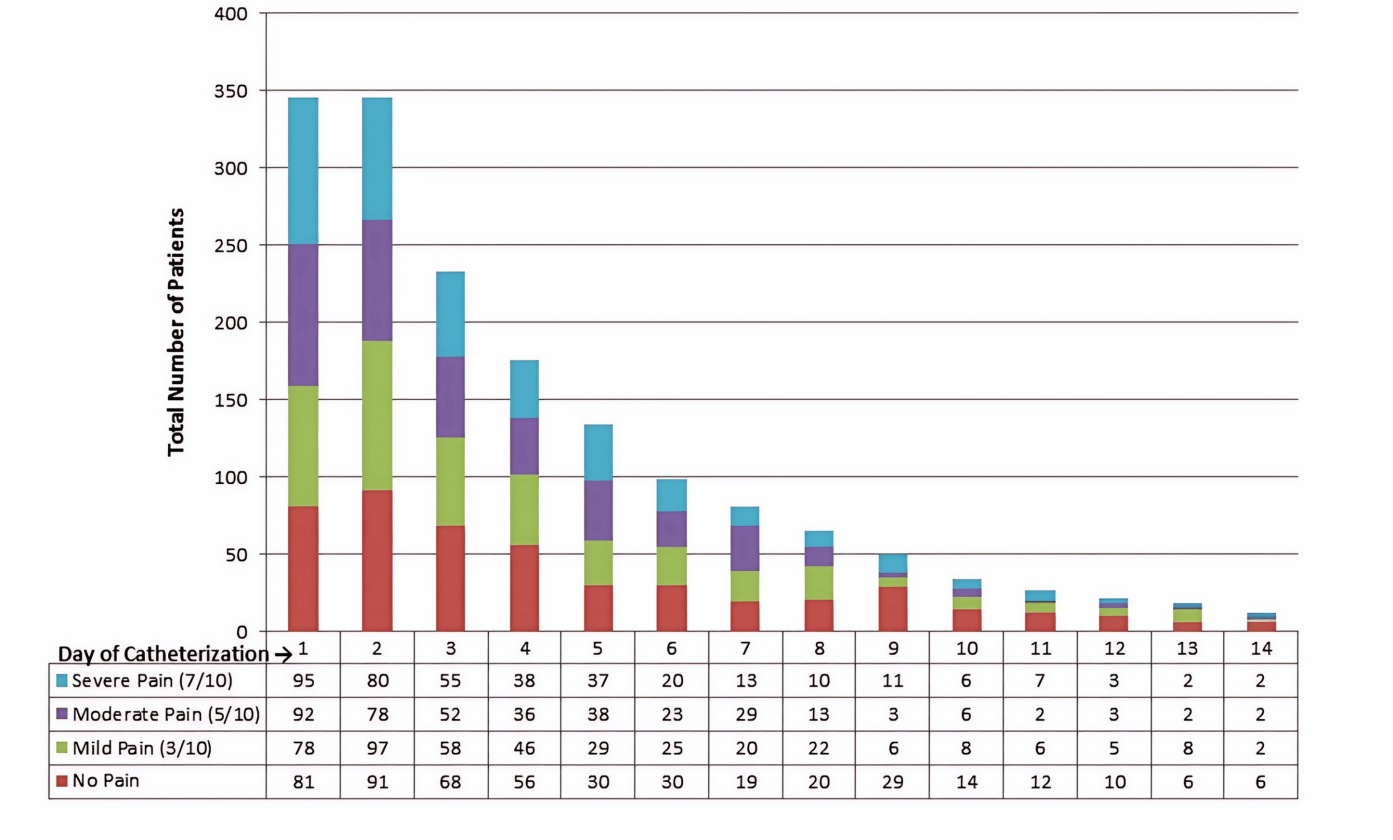
Distribution of Suprapubic Pain Intensity This figure demonstrates changes in suprapubic pain intensity, measured using the Visual Analogue Scale (VAS), during 14 days of catheterization. The proportion of patients reporting severe pain decreased from 27.5% on Day 1 to 16.7% on Day 14, while moderate pain decreased from 26.6% to 16.7%. In contrast, the proportion of patients reporting no pain increased from 23.4% to 50% during the same period. These findings indicate a progressive reduction in pain intensity over time, which may reflect ongoing symptom management and patient adaptation to catheterization. VAS: Visual Analogue Scale

Pain intensity, assessed using the visual analogue scale (VAS), declined progressively across the study period. The proportion of patients with severe pain decreased from 27.5% on day one to 16.7% on day 14, while moderate pain decreased from 26.6% to 16.7 (Figure 3). In contrast, the proportion of patients reporting no pain increased from 23.4% to 50% during the same period. These descriptive findings indicate a general reduction in pain intensity over time, which may reflect the effect of ongoing symptom management and patient adaptation to catheterization.

CAUTI rate per 1,000 catheter days

CAUTI rates were comparable between groups. The clean group had 13 infections over 692 catheter days, while the sterile group had 12 infections over 519 catheter days. Overall, 25 infections occurred across 1,211 catheter days. 

CAUTI rate = 25/346 × 100 = 7.23% 

Incidence rate = 25/1211 × 1000 = 20.64 per 1000 catheter days

Microbiological flora and antibiogram

**Figure 4:**
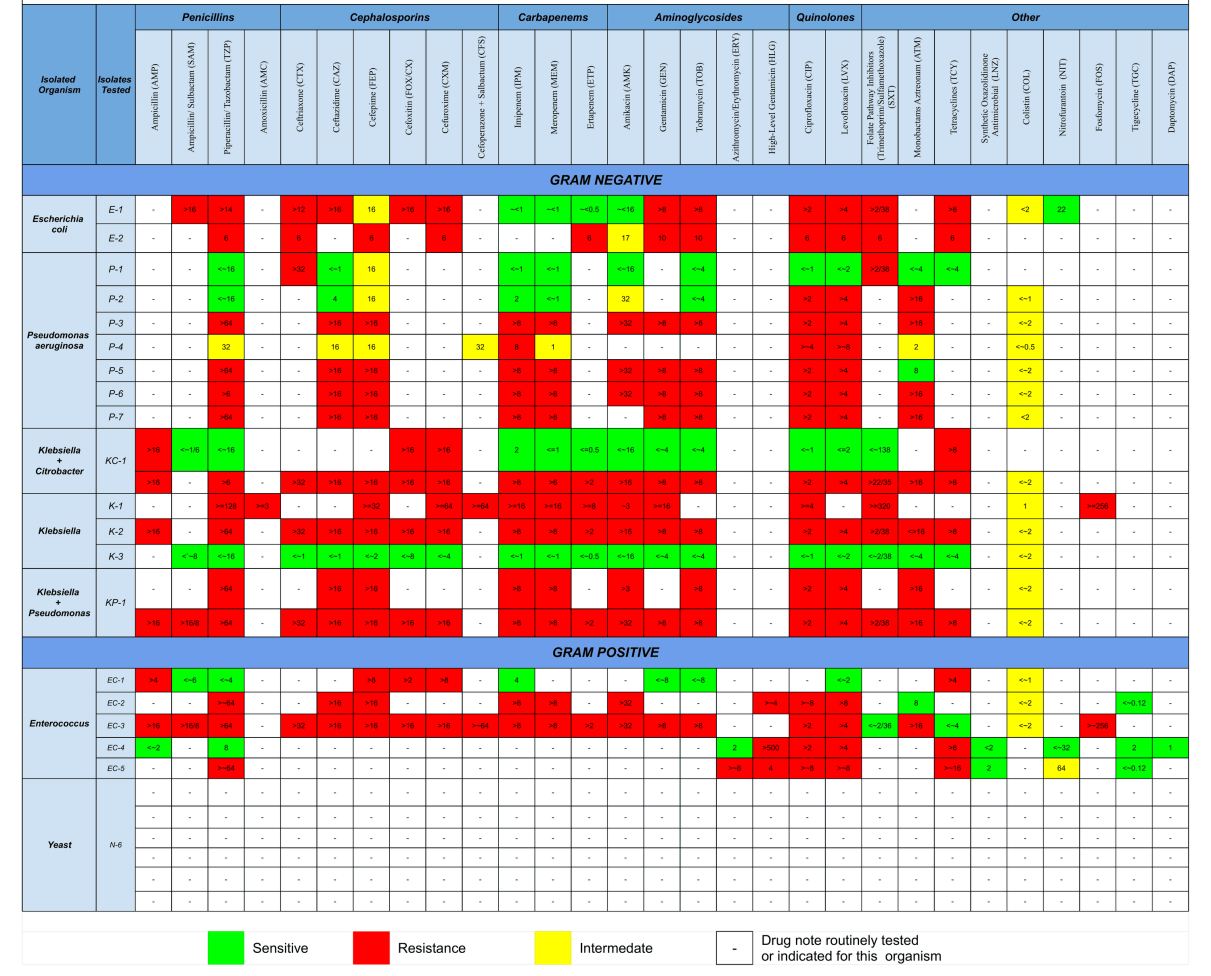
Urinary Isolated Organism Antibiogram This figure shows the antibiogram of urine culture isolates (n = 25) from CAUTI patients. Susceptibility testing was performed using automated MIC-based methods and interpreted by CLSI breakpoints as Sensitive (S), Intermediate (I), Resistant (R), or Susceptible Dose Dependent (SDD). The chart highlights organism-wise resistance and susceptibility patterns. Escherichia coli and Pseudomonas aeruginosa demonstrated resistance to cephalosporins and fluoroquinolones but remained sensitive to carbapenems and colistin; Klebsiella pneumoniae showed marked resistance to beta-lactams with retained carbapenem susceptibility; Enterococcus species were resistant to penicillins but susceptible to linezolid; and several Candida/yeast isolates were also identified. Colors indicate susceptibility categories for ease of interpretation. MIC: Minimum Inhibitory Concentration; CAUTI: Catheter-Associated Urinary Tract Infection

A total of 25 organisms were isolated from urine cultures. The antibiogram results, expressed as MIC values and interpreted into S/I/R/SDD categories, were summarized descriptively (Figure 4). Escherichia coli and Pseudomonas aeruginosa isolates demonstrated high resistance to cephalosporins and fluoroquinolones but remained sensitive to carbapenems and colistin. Klebsiella pneumoniae isolates showed marked resistance to beta-lactams but were generally susceptible to carbapenems. Enterococcus species were resistant to penicillins but retained susceptibility to linezolid. In addition, several Candida/yeast isolates were recovered.

## Discussion

This study suggests that clean catheter care may be a feasible alternative to sterile catheter care in preventing CAUTI, particularly in resource-limited settings. When performed with adherence to infection control measures, clean catheter care did not result in a significantly higher CAUTI incidence compared with sterile care. The findings also reinforce the importance of timely catheter removal and minimizing catheter duration.

Effectiveness of clean vs. sterile catheter care

CAUTI incidence was 7.5% in the clean group and 6.9% in the sterile group (p = 0.83), with an overall incidence of 20.64 per 1,000 catheter days. After excluding fungal infections, the adjusted incidence decreased to 15.69 per 1,000 catheter days. Prolonged catheterization was the only independent factor significantly associated with CAUTI, consistent with prior evidence that the duration of catheterization is the strongest modifiable risk factor [1,4,6]. Several previous studies have demonstrated a stepwise increase in bacteriuria and CAUTI risk with each additional day of catheterization, underscoring the importance of daily reassessment and early catheter removal strategies [9,16,18,20,21].

Symptom trends and patient experience

Daily monitoring documented fluctuating symptom patterns. Pain prevalence declined from 77% on Day 1 to 50% on Day 14, while severe pain decreased from 27.5% to 16.7%. Fever peaked mid-course, and agitation/confusion appeared later. These findings highlight the importance of structured symptom surveillance and early recognition of discomfort and neurocognitive changes in catheterized patients, in line with prior reports emphasizing symptom burden during prolonged catheterization [12].

Microbiological and antibiotic considerations

Urine cultures demonstrated a range of MDR organisms, including cephalosporin- and fluoroquinolone-resistant isolates, though carbapenems and colistin remained effective. *Enterococcus* showed penicillin resistance but susceptibility to linezolid. Several Candida/yeast isolates were also identified. Antibiotics were prescribed in both groups strictly according to clinical indications, not influenced by study assignment. However, antibiotic exposure was not systematically adjusted for in the analysis, which may have influenced outcomes. This finding underscores the importance of meticulous catheter care and minimizing catheter duration, as highlighted in prior antimicrobial resistance studies [9-13].

Clinical implications

The results have practical implications for healthcare systems where sterile catheter care may not always be feasible. Clean catheter care, when standardized and supervised, appears non-inferior in safety and effectiveness. This aligns with international guidelines supporting clean catheter care for maintenance, particularly in community and home settings [4-8]. For patients discharged with catheters in situ, clean catheter care is also feasible at home, further supporting its sustainability in diverse healthcare environments.

Limitations

This study has several important limitations. First, although the sample size was calculated a priori, the relatively small number of CAUTI events meant the study was underpowered, and attrition may have further reduced effective power. Second, catheter care in both groups was delivered by the same pool of nursing officers. Despite standardized training and spot checks, complete intervention fidelity could not be ensured, and some degree of crossover bias may have occurred. Third, analyses were performed per protocol rather than intention-to-treat, which may have introduced selection bias. Fourth, although logistic regression adjusted for age, gender, and catheter duration, antibiotic exposure could not be consistently captured and therefore was not included, limiting adjustment for this important confounder. Fifth, while participants and microbiologists were blinded, healthcare providers were not. Sixth, urine cultures were obtained only at baseline and at predefined clinical endpoints, so asymptomatic bacteriuria was not assessed. Seventh, the study was conducted at a single center, which limits generalizability to broader populations. Finally, a potential Hawthorne effect cannot be ruled out, as staff awareness of being observed might have enhanced adherence to care protocols in both groups.

## Conclusions

This randomized controlled trial found that clean catheter care, when performed with strict adherence to infection control protocols, was non-inferior to sterile catheter care in preventing CAUTI. Prolonged catheterization remained the most significant independent risk factor for infection. These findings highlight that clean catheter care is an effective and practical alternative to sterile care, particularly within resource-limited healthcare settings. It also makes it easier for patients to engage in self-care while managing indwelling catheters at home. Emerging innovations such as visible light-based disinfection systems and antimicrobial-coated indwelling urinary catheters represent promising adjuncts to conventional infection-prevention strategies. Integrating such technologies with standardized clean catheter-care protocols may further strengthen infection control and enhance patient safety.

## Appendices

Supplementary appendix 1

Protocols for Sterile and Clean Indwelling Catheter Care

**Table 3:** Procedure for Sterile Versus Clean Catheter Care Technique

Step	Sterile Technique	Clean Technique
Definition	Cleaning catheter and insertion site using sterile gloves, sterile gauze, artery forceps, and strict aseptic precautions	Cleaning catheter and insertion site using clean gloves and gauze under hygienic precautions
Purpose	• Promote patient comfort • Reduce risk of CAUTI	• Practical and sustainable for patients/caregivers • Reduce risk of CAUTI
Articles Required	• Sterile gloves • Sterile gauze • Artery forceps • Kidney tray/bowl • Normal saline (0.9%) • Sterile sheet/drape • Mackintosh • Adhesive tape • Alcohol-based hand rub	• Clean gloves • Clean gauze • Normal saline (0.9%) • Plain sheet • Mackintosh • Adhesive tape • Alcohol-based hand rub
1. Preparation	Perform hand hygiene, prepare sterile supplies	Perform hand hygiene, prepare clean supplies
2. Patient Communication	Explain procedure, provide privacy	Explain procedure, provide privacy
3. Positioning	Female: dorsal recumbent with legs flexed Male: supine	Female: dorsal recumbent with legs flexed Male: supine
4. Draping	Mackintosh + sterile sheet	Mackintosh + plain sheet
5. Catheter Access	Remove adhesive tape, maintain sterile field	Remove adhesive tape
6. Gloves	Wear sterile gloves	Wear clean gloves
7. Exposure	Use non-dominant hand to retract labia (female) or foreskin (male)	Use non-dominant hand to retract labia (female) or foreskin (male)
8. Assessment	Check for inflammation, swelling, discharge	Check for inflammation, swelling, discharge
9. Perineal Cleansing	Sterile gauze + saline: • Female: labia majora → labia minora → urethral meatus (front to back) → vaginal orifice (if needed) • Male: clean urethral meatus first (circular motion outward) → shaft → return foreskin	Clean gauze + saline: • Female: labia majora → labia minora → urethral meatus (front to back) → vaginal orifice (if needed) • Male: clean urethral meatus first (circular motion outward) → shaft → return foreskin
10. Catheter Cleansing	Wipe 3–5 cm outward from meatus with sterile gauze soaked in saline	Wipe 3–5 cm outward from meatus with clean gauze soaked in saline
11. Re-securing	Re-secure catheter with adhesive tape	Re-secure catheter with adhesive tape
12. Completion	Reposition patient, ensure comfort	Reposition patient, ensure comfort
13. Disposal	Dispose of contaminated supplies, perform hand hygiene	Dispose of contaminated supplies, perform hand hygiene
14. Documentation	Record procedure, complete CAUTI bundle checklist	Record procedure, complete CAUTI bundle checklist

Supplementary appendix 2

Urine Culture Collection Technique for Catheterized Patients

**Table 4:** Urine Specimen Collection Procedure in Catheterized Patients

Step	Procedure	Notes/Precautions
1	Explain the procedure to the patient	Ensure cooperation and safety
2	Perform hand hygiene and apply gloves	Maintain aseptic technique
3	Clamp catheter tubing below the sampling port for 15–30 minutes	Allows urine to collect; avoid >30 minutes
4	Disinfect the sampling port with 70% isopropyl alcohol and allow to dry	Prevent contamination
5	Using a sterile syringe, aspirate 5–10 mL urine from the sampling port	Do not collect from drainage bag
6	Transfer urine into a sterile container without touching inside surfaces	Maintain sterility
7	Label specimen with patient details, date, and time	Prevent misidentification
8	Unclamp catheter tubing	Restore urine drainage
9	Transport specimen to microbiology laboratory within 2 hours	If delay, refrigerate at 2–8 °C, maximum 24 hours
